# An instrumental variable random-coefficients model for binary outcomes

**DOI:** 10.1111/ectj.12018

**Published:** 2014-04-22

**Authors:** Andrew Chesher, Adam M Rosen

**Affiliations:** † Centre for Microdata Methods and Practice, Institute for Fiscal Studies7 Ridgmount Street, London, WC1E 7AE, UK; ‡ Department of Economics, University College LondonGower Street, London, WC1E 6BT, UK

**Keywords:** Endogeneity, Incomplete models, Instrumental variables, Partial identification, Random coefficients, Random sets, Set identification

## Abstract

In this paper, we study a random-coefficients model for a binary outcome. We allow for the possibility that some or even all of the explanatory variables are arbitrarily correlated with the random coefficients, thus permitting endogeneity. We assume the existence of observed instrumental variables *Z* that are jointly independent with the random coefficients, although we place no structure on the joint determination of the endogenous variable *X* and instruments *Z*, as would be required for a control function approach. The model fits within the spectrum of generalized instrumental variable models, and we thus apply identification results from our previous studies of such models to the present context, demonstrating their use. Specifically, we characterize the identified set for the distribution of random coefficients in the binary response model with endogeneity via a collection of conditional moment inequalities, and we investigate the structure of these sets by way of numerical illustration.

## 1. INTRODUCTION

In this paper, we analyse a random-coefficients model for a binary outcome,
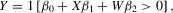
(1.1)where 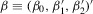 are random coefficients. Although covariates *W* are restricted to be exogenous, covariates *X* are permitted to be endogenous in the sense that the joint distribution of *X* and random coefficients β is not restricted. We assume that in addition to the variables 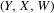, the researcher observes realizations of a random vector of instrumental variables *Z* such that 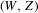 and β are independently distributed. Thus, our goal is to use knowledge of the joint distribution of 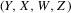 to set identify the marginal distribution of the random coefficients β, denoted 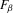, with the joint distribution of random vectors *X* and β left unrestricted. As a special case, we also allow for the possibility there are no exogenous regressors *W*.^1^ As shorthand, we use the notation 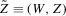 to denote the composite vector of all exogenous variables.

In order to characterize the identified set for 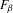, we carry out our identification analysis along the lines of Chesher et al. (2013), hereafter CRS, and Chesher and Rosen et al. (2013). Like CRS, we consider a single-equation model for a discrete outcome, but here we restrict the outcome to be binary. However, the model (1.1) used in this paper features random coefficients, which are not present in CRS. The model is a special case of the general class of models considered in Chesher and Rosen et al. (2013), where we provide identification analysis for a broad class of instrumental variable (IV) models. Like those models, the random-coefficients model (1.1) allows for multiple sources of unobserved heterogeneity whereas, traditionally, IV methods have been employed in models admitting a single source of unobserved heterogeneity. Thus, in this paper, we investigate, and illustrate by way of example, the identifying power of IV restrictions with multivariate unobserved heterogeneity in the determination of a binary outcome. The characterizations we employ rely on results from random set theory. These and related results have been used for identification analysis in various ways and in a variety of contexts by Beresteanu et al. (2011, 2012), Galichon and Henry et al. (2011), CRS, and Chesher and Rosen (2012, 2013). As in CRS and Chesher and Rosen (2012, 2013), our characterizations make use of properties of conditional distributions of certain random sets in the space of unobserved heterogeneity.

The model also builds on the IV models for binary outcomes considered in Chesher (2010, 2013), where a single source of unobserved heterogeneity was permitted. There, it was found that even if parametric restrictions were brought to bear, the models were in general not point identifying. So, with the addition of further sources of unobserved heterogeneity, point identification should not generally be expected. The paper thus serves to illustrate in part the effect of additional sources of heterogeneity from the perspective of identification. The case of a binary outcome variable is convenient for illustration, but models that permit more variation in outcome variables might achieve greater identifying power.

Binary response specifications that model β in (1.1) as a random vector include, for example, those of Quandt et al. (1966) and McFadden et al. (1976), and can be viewed as special cases of the discrete choice models of Hausman and Wise et al. (1978) and Lerman and Manski et al. (1981). These papers focus on specifications where all covariates and β are independently distributed, and where the distribution of β is parametrically specified, enabling estimation via maximum likelihood. Ichimura and Thompson et al. (1998) and Gautier and Kitamura et al. (2013) focus on the binary outcome model (1.1), again with covariates and random coefficients independently distributed, but with 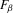 non-parametrically specified. Ichimura and Thompson et al. (1998) provide sufficient conditions for point identification of 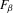 in this case, and prove that 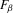 can be consistently estimated via non-parametric maximum likelihood. Gautier and Kitamura et al. (2013) introduce a computationally simple estimator for the density of β, and derive its rate of convergence and pointwise asymptotic normality. Gautier and LePennec et al. (2011) propose an adaptive estimation method.

In contrast, we do not require that 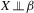 and we employ instrumental variables *Z*. The use of an IV approach in a random-coefficients binary response model with endogeneity is new. A control function approach is employed by Hoderlein et al. (2009) to provide identification results for marginal effects and local average structural derivatives when a triangular structure is assumed for the determination of *X* as a function of *Z*. Hoderlein and Sherman et al. (2011) study identification and estimation of a trimmed mean of random coefficient β when again endogenous variables can be written as a function of mutually independent instruments *Z* and control variables *V*, additionally employing some conditional median restrictions. However, our model does not require one to specify the form of the stochastic relation between *X* and *Z*, and is thus incomplete.^2^

The random-coefficients logit model of Berry et al. (1995), hereafter BLP, now a bedrock of the empirical industrial organization literature, allows for endogeneity of prices using insight from Berry et al. (1994) to handle endogeneity. Yet, the endogeneity problem in that and related models in industrial organization is fundamentally different from the one in this paper. Their approach deals with correlation between alternative-specific unobservables with prices at the market level, both of which are assumed independent of random coefficients that allow for consumer-specific heterogeneity. Important identification results in such models are provided by Berry and Haile (2009, 2010), and a general treatment of the literature on such models and their relation to other models of demand is given by Nevo et al. (2011). Here, we focus on binary response models at a micro-level, rather than across separate markets, absent alternative-specific unobservables, and we allow random coefficients to be correlated with regressors.^3^ Recent papers that give identification results for micro-level discrete choice models with exogenous covariates and high-dimensional unobserved heterogeneity include Briesch et al. (2010), Bajari et al. (2012), and Fox and Gandhi et al. (2012). The latter also allows for endogeneity with alternative-specific special regressors and further structure on the determination of endogenous regressors as a function of the instruments.

The paper is organized as follows. In Section 2, we formally present our model and key restrictions, and we introduce a simple example in which there is one endogenous regressor and no exogenous regressors. In Section 3, we characterize the identified set for the distribution of random coefficients in the general model set out in Section 2, and we provide two further examples. In Section 4, we provide numerical illustrations of identified sets for subsets of parameters in a parametric version of our model for four different data-generation processes. We conclude in Section 5. The proof of the main identification result, which adapts theorems from CRS, is provided in Appendix 1. Appendix B provides computational details absent from the main text, and Appendix C verifies that there would be point identification in the example considered in the numerical illustrations of Section 4 if exogeneity restrictions were imposed.

Throughout the paper, we use the following notation. We use upper-case Roman letters *A* to denote random variables and lower-case letters *a* to denote particular realizations. For the probability measure 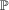, 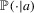 is used to denote the conditional probability measure given 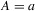. The calligraphic font 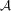 is used to denote the support of *A* for any well-defined random variable *A* in our model. 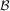 denotes the support of the random-coefficients vector β, and 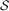 denotes a closed set on 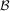. For any pair of random vectors 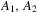, 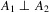 denotes stochastic independence, Supp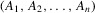 denotes the joint support of the collection of random vectors 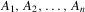, and Supp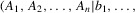 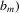 denotes the conditional support of 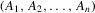 given realizations 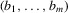 of random vectors 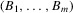. The empty set is denoted by ∅. We use 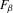 to denote the probability distribution of β, mapping from subsets of 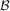 to the unit interval. 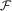 is used to denote the admissible parameter space for 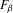, *F* is used to denote a generic element of 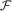, and 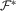 denotes the identified set for 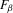. We use cl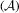 to denote the closure of a set 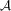. Finally, 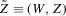 with support denoted 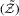 is used to denote the vector of all exogenous variables, and 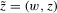 for particular realizations.

## 2. THE MODEL

We now formally set out the restrictions of our model.

Restriction 2.1. 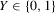, 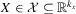, and 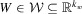 obey (1.1) for some unobserved 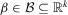 with 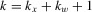, and 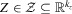. 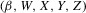 belong to a probability space 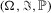 endowed with the Borel sets on Ω and the joint distribution of 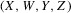, denoted 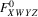, is identified. For all 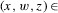 Supp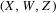, 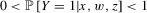.

Restriction 2.2 For any 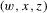 on the support of 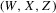, the conditional distribution of random vector β given 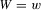, 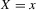, and 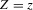 is absolutely continuous with respect to Lebesgue measure on 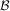. β is marginally distributed according to the probability measure 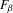 mapping from subsets of 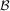 to the unit interval, with associated density 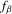. 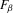 is known to belong to some class of probability measures 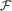.^4^

Restriction 2.3 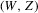 *and β are independently distributed*.

Restriction 2.1 invokes the random-coefficients model for the binary outcome *Y* and defines the support of random vectors *X*, *W*, and *Z*. The restriction further requires that for all 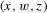, both 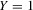 and 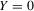 have positive probability 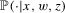. This simplifies the exposition of some of the developments that follow, but is not essential. We do not otherwise restrict the joint support of 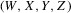. We require that the joint distribution of 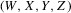 is identified, as would be the case under random sampling, for instance. Restriction 2.3 is our IV restriction, requiring independence of 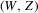 and β. Restriction 2.2 restricts 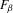 to some known class of distribution functions. In principle, this class could be parametrically, semi-parametrically, or non-parametrically specified. Of course, greater identifying power will be afforded when 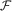 is parametrically specified. In our numerical illustrations in Section 4, β is restricted to be normally distributed, which is a common restriction in random-coefficients models.

As is always the case in models of binary response, it will be prudent to impose a scale normalization because 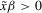 holds if and only if 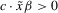 for all scalars 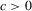, where 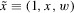.^5^ This can be done by imposing, for example, that 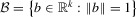 if 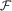 is non-parametrically specified, or by imposing that the first component of β has unit variance (e.g., when 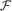 is parametrically specified as in the following example, and as also employed in the numerical illustrations of Section 4).

Example 2.1 One endogenous variable, no exogenous variables Suppose *X* ∈ 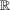 and that there are no exogenous covariates *W*. Then, we can write (1.1) as
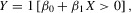
with 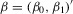. Suppose that 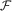 is the class of bivariate normal distributions whose first component has unit variance. Then, defining α_0_, α_1_ as the means of β_0_, β_1_, respectively, we have the representation
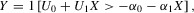
(2.1)where 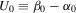 and 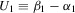 are mean-zero bivariate normally distributed with the same variance as 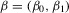. We then have from Restriction 2.3 that 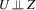, and we can parametrize the distribution 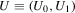 as
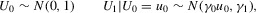
equivalently
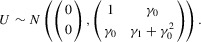
(2.2)Knowledge of the parameter vector 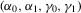 would then suffice for the determination of 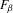, so the identified set for 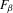 can be succinctly expressed as the identified set for 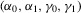.

## 3. IDENTIFICATION

For identification analysis, it will be useful to consider the correspondence
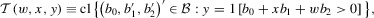
(3.1)which is the closure of the halfspace of 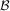 on which 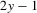 and 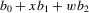 have the same sign. Application of this correspondence to random elements 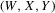 yields a random closed set 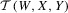. For any realization of the exogenous variables 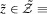 Supp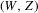, the conditional distribution of this random set given 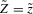 is completely determined by the distribution of 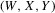 given 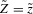, which is identified given knowledge of 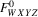 under Restriction 2.2. The identified set for 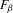, denoted 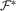, is then the set of measures 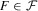 that are selectionable from the conditional distribution of 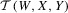 given 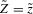 for almost every 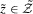.^6^ Intuitively, this holds because selectionability guarantees the existence of a random variable 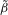 realized on 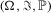 and distributed *F*, such that 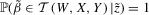, a.e. 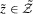.^7^ Thus, there exists a random variable 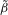 distributed *F* that delivers the conditional distribution
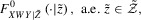
and all such *F* are observationally equivalent.

As done in CRS for utility-maximizing discrete choice models without random coefficients and in Chesher and Rosen et al. (2013) for single-equation IV models more generally, we can exploit Artstein's Inequality (Artstein, 1983, see also Norberg, 1992, and Molchanov, 2005, Section 1.4.8.) to characterize the identified set through the use of conditional containment functional inequalities. Using the same steps taken in Theorem 1 of CRS, Artstein's Inequality guarantees that a distribution *F* is selectionable from the conditional distribution of 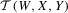 given 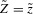, if and only if for all closed sets 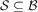,
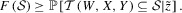
(3.2)

The use of the conditional containment inequality (3.2) reduces the problem of determining which *F* are selectionable from 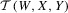 to a collection of conditional moment inequalities. In CRS and Chesher and Rosen (2012, 2013), we devised algorithms to determine which test sets 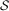 are sufficient in the contexts of the models in those papers to imply (3.2) for all possible test sets 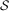. The collection of such sets, referred to as core-determining sets, is crucially dependent on the support of the random set under consideration. By the same reasoning as in those papers, it is sufficient to focus on test sets that are unions of sets that belong to the support of 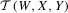 conditional on the realization of exogenous variables 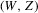. For any such realization 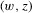, the support of 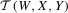 is the collection of sets
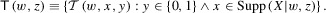
(3.3)We do not require that the conditional support of *X* given 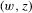 coincide with its unconditional support, but in that case Supp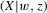 in (3.3) can be replaced with 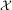, and the collection of sets 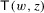 does not vary with 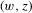. The larger the conditional support Supp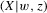, the larger the core-determining collection of test sets will be.

Given any 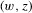, each element of 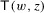 is a halfspace in 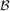, so the required test sets 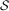 take the form of unions of such halfspaces.^8^ Alternatively, each such test set can be written as the complement of intersections of sets, each of which are complements of elements of 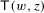. This is convenient because the complement of each 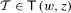, denoted 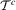, is also a halfspace, and the intersection of halfspaces is a convex polytope. Thus, the collection of core-determining test sets 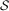 contains sets that are complements of intersections of halfspaces, equivalently complements of convex polytopes. The formal result follows.

Theorem 3.1 *Let Restrictions 2.1–2.3 hold. Then, the identified set for* 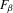 *is*
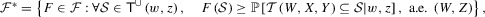
(3.4)where 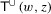 denotes the collection of sets that are unions of members of 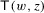. Equivalently,
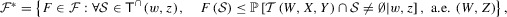
(3.5)where 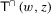 denotes the collection of sets that are intersections of members of 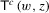, where
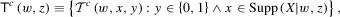
which is the collection of sets that are complements of those in 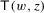.

The theorem follows from consideration of Theorems 1 and 2 of CRS, adapted to the random set 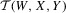 defined in (3.1), which make use of Artstein's Inequality (Artstein, 1983) to prove sharpness; see also Norberg et al. (1992) and Molchanov et al. (2005), Section 1.4.8. The characterization of test sets for the containment functional characterization (3.4) of Theorem 2 in CRS stipulates that a core-determining collection of test sets 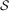 is given by those that are (i) unions of elements of 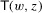, and (ii) such that the union of the interiors of component sets is a connected set. In this paper, condition (ii) can be ignored because the sets 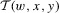 and 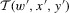 are all halfspaces through the origin, ensuring that 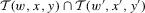 has open interior except in the special case 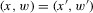 and 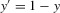, in which case 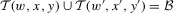. The test set 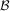 can indeed be safely discarded from consideration because from 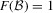, (3.4) is trivially satisfied. The equivalence of the containment functional characterization (3.4) and the capacity functional characterization (3.5) follow from the fact that, for any sets 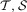, the events 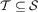 and 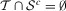 are identical.

Theorem 3.1 provides a characterization of the identified set of distributions of random coefficients for binary choice models with endogeneity and instrumental variables. In particular, the representation is given by a collection of conditional moment inequalities, with one such inequality conditional on the realization of exogenous variables 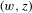 for each element of 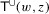 in (3.4), equivalently one conditional moment inequality for each element of 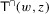 in (3.5). These conditional moment inequalities can then be used as a basis for estimation and inference. To illustrate, suppose that the endogenous variable *X* is discrete, so that for any 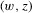, 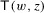 is a finite collection of sets in 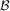. We can therefore enumerate the elements of 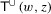 as 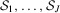 for some 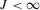. Suppose further that 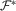 is parametrically specified up to finite-dimensional parameter θ, with typical element 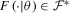. The characterization of the identified set in (3.4) can then be written as those 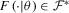 such that
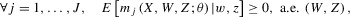
where
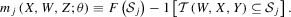


Inference can then be based on these conditional moment inequalities using, for example, methods from Andrews and Shi et al. (2013) or Chernozhukov et al. (2013).

In some important special cases, considered in the following examples, characterization of the identified set can be further simplified.

Example 3.1. No endogenous covariates A leading and well-studied example is the case where there are no endogenous variables *X*. Then, for each 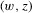, we have
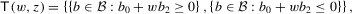
where *b* is of the form 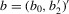. The intersection of these sets is 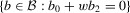, which has zero measure 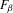 under Restriction 2.2, and their union is 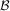, which has measure 1. It follows from similar reasoning as in Theorem 6 of Chesher and Rosen et al. (2012) that for any 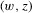 the inequalities of the characterizations of Theorem 3.1 produce moment equalities. Consider, for example, the containment functional inequalities of (3.4) delivered by all 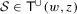:
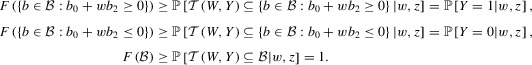
The last inequality is trivially satisfied for all 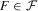. Both the right-hand sides and the left-hand sides of the first two inequalities clearly sum to 1, implying that these inequalities must, in fact, hold with equality, giving
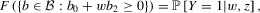
(3.6)
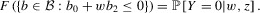
(3.7)When there are no excluded exogenous variables *z* and 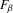 is not restricted to a parametric family, these equations coincide with the identifying equations in Ichimura and Thompson et al. (1998) and Gautier and Kitamura et al. (2013). Ichimura and Thompson et al. (1998) provide sufficient conditions for point identification.^9^ When *F* is parametrically restricted, these equalities are likelihood contributions (e.g., integrals with respect to the normal density in Hausman and Wise, 1978 or Lerman and Manski, 1981), and less stringent conditions are required for point identification. In the absence of sufficient conditions for point identification, the moment equalities (3.6) and (3.7) a.e. 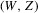 nonetheless fully characterize the identified set.

Example 3.2. One endogenous covariate with arbitrary exogenous covariates Consider the common setting where there is a single endogenous explanatory variable, 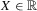, as well as some exogenous explanatory variables *W*, a random 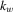-vector. Then, given any 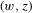, the collection of sets 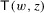 is given by
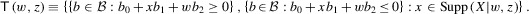
Suppose, for simplicity, that Supp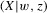 is discrete. Consider now a test set 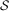 which is one of the core-determining sets in 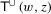 and hence an arbitrary union of sets in 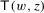.^10^ Any such 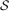 can be equivalently written as the set of 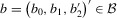 that satisfy one of the inequalities
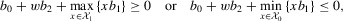
(3.8)for some collections of values 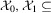 Supp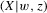.

Define now for each 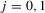,
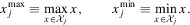
If 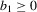, (3.8) simplifies to
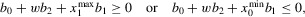
(3.9)while if 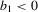, the inequalities can be written
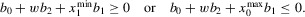
(3.10)Furthermore, for any 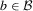 with 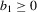, (3.10) implies (3.9), and for any 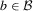 with 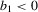, (3.9) implies (3.10). Thus, for any 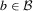, (3.8) holds if and only if
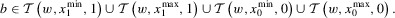
From this, it follows that one need only consider for each 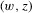 test sets 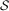 of the form
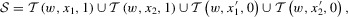
where 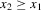 and 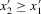.

Example 2.1 Continued If we restrict attention to cases with no exogenous covariates *W*, there is in fact further simplification of the list of core-determining sets. To see why, note that in this case the collection 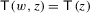 for any *z* reduces to
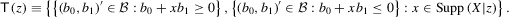
Each element of 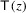 is thus a halfspace in 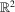 defined by a separating hyperplane through the origin intersected with 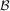. The union of an arbitrary number of such halfspaces can be equivalently written as the union of no more than two such halfspaces. Therefore, the collection of core-determining sets 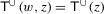 is given by the collection of test sets that can be written as either elements of 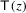 or unions of a pair of elements in 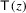,
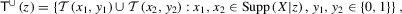
(3.11)where for any 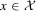 and 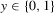,
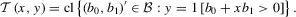
The characterization applies for either continuous or discrete *X*, but if *X* is discrete with *K* points of support, there are no more than 2*K*^2^ sets in 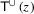 for any 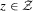. This follows from noting there are 2*K* unique 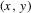 pairs and the number of all pairwise unions (including the union of each set with itself) is 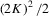, with division by two from the observation that for any 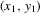 and 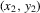, 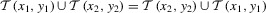.

In the numerical illustrations that follow we consider various instances of Example 2.1, where there are no exogenous covariates *W* and where *F* is restricted to a parametric (specifically Gaussian) family. In the illustrations, we investigate identified sets for averages of 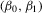, and we show that this affords further computational simplification, in the sense that for any fixed candidate values of 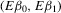, we need only consider test sets 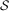 that are unions of two elements of 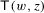 in order to check whether such candidate values belong to the identified set.

## 4. NUMERICAL ILLUSTRATIONS

To investigate the identifying power of the binary outcome random-coefficients IV model, we reconsider Example 2.1. *Y* is determined as in (2.1) with unobservable 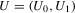 bivariate normal with zero mean and variance as parametrized in (2.2). We define
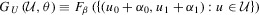
as the probability that *U* belongs to the set 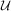 where 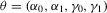 and when β is distributed 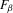 with mean α and variance governed by parameters 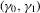. Given the restriction that 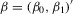 is bivariate normally distributed, knowledge of θ implies knowledge of 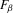. Thus, we consider the identified set for θ, denoted 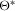, and focus attention on the identified set for 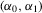, the projection of the first two elements of 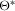 on 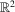.

### 4.1 Data-generating processes

Our examples employ data-generating processes in which *X* is determined as follows:
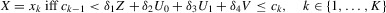
(4.1)
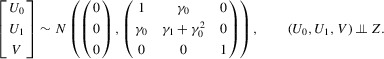
We report four calculations, in all of which there are the following settings:
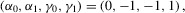

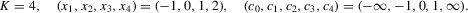


In two cases (N1 and N2), the parameters are set such that *X* is endogenous, and in another two cases (X1 and X2), they are set such that *X* is exogenous. We consider two possibilities for the coefficient δ_1_ multiplying instrument *Z* in the determination of *X* in (4.1): 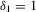 (N1 and X1) and 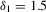 (N2 and X2). All parameter settings are shown in Table 1. Table 2 shows the two conditional distributions of *X* given *Z*. In all cases, the support of the instrumental variable is 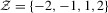.

**Table 1:** Parameter settings in the four calculations

	Endogenous X		Exogenous X	
Parameter	Case N1	Case N2	Case X1	Case X2
δ_1_	1.000	1.500	1.000	1.500
δ_2_	0.577	0.577	0.000	0.000
δ_3_	−0.577	−0.577	0.000	0.000
δ_4_	0.577	0.462	1.414	1.371

**Table 2:** Conditional probabilities 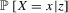

		*z* = −2	*z* = −1	*z* = +2	*z* = +2
δ_1_= 1.0	*x* = −1	0.760	0.500	0.079	0.017
	*x* = 0	0.161	0.260	0.161	0.062
	*x* = 1	0.062	0.161	0.260	0.161
	*x* = 2	0.017	0.079	0.500	0.760
δ_1_= 1.5	*x* = −1	0.928	0.642	0.034	0.002
	*x* = 0	0.058	0.221	0.103	0.013
	*x* = 1	0.013	0.103	0.221	0.058
	*x* = 2	0.002	0.034	0.642	0.928

If the exogeneity restriction 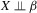 is imposed then, as shown in Appendix C, the resulting model point identifies the full parameter vector θ. In the structures delivering probability distributions in cases X1 and X2, it is the case that 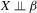 holds. However, we calculate identified sets for a model without the exogeneity restriction and thereby show the substantial loss in identifying power arising when exogeneity cannot be assumed to hold.

### 4.2 Calculation of probabilities

To illustrate identified sets, we computed the conditional probabilities 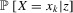 and 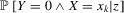. 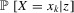 is given by
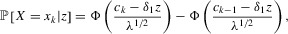
where 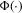 denotes the standard normal distribution function and
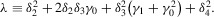
(4.2)The conditional probability 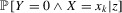 can be calculated as the difference between two normal orthant probabilities because, when 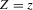, we have
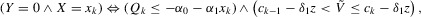
where
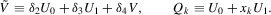
Because 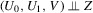, conditional on 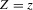, we have
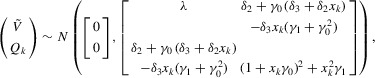
from which we see that 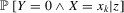 is indeed the difference between two normal orthant probabilities. The conditional probability 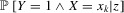 can then be obtained by subtracting 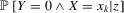 from 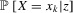.^11^

### 4.3 Calculation of projections

We calculate two-dimensional projections of the four-dimensional (4D) identified set for θ_0_, giving results for the projection on to the plane on which lie 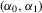. This is the identified set for the mean of the random coefficients 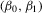.

We calculate the projections as follows.^12^ The full 4D identified set is
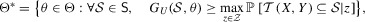
(4.3)where 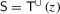 is a collection of 32 core-determining sets of the form described for Example 2.1 in Section 3, specifically (3.11), in the present case where *X* has four points of support. 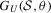 is the probability mass placed on the set 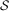 by a bivariate normal distribution with parameters θ. The probabilities 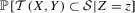, 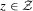, are identified under Restriction 2.1.

For computational purposes, we make use of the following discrepancy measure
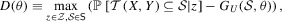
(4.4)which can be used to characterize the full 4D identified set as follows:
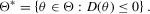


To compute identified sets for subvectors of parameters, let 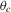 denote a list of one or more elements of θ, and let 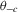 denote the remaining elements of θ. The projection of the identified set on to the space in which 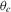 resides is the set of values of 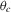 for which there exists 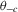 such that 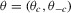 lies in the identified set 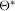. We calculate this set, 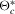, as the set of values 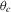 for which the value of 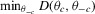 is non-positive:
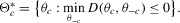
(4.5)Here, 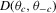 is to be understood as the function defined in (4.4) applied to that value of θ with subvectors equal to 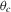 and 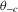. We perform this minimization using the optimfunction in base R.

**Figure 1:**
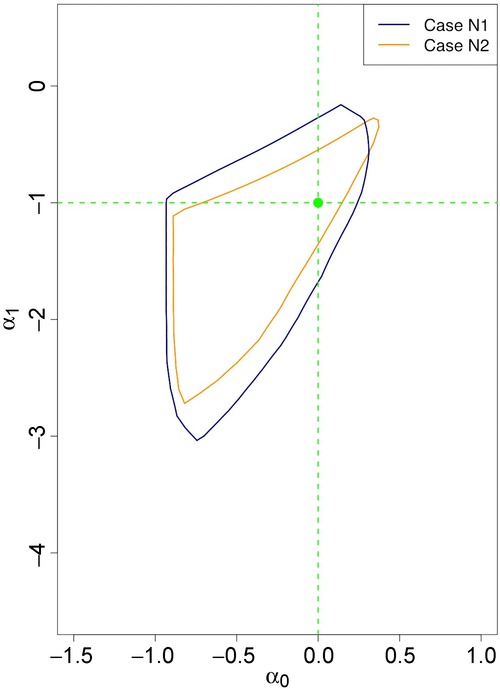
Projections of identified sets for cases N1 and N2.

**Figure 2:**
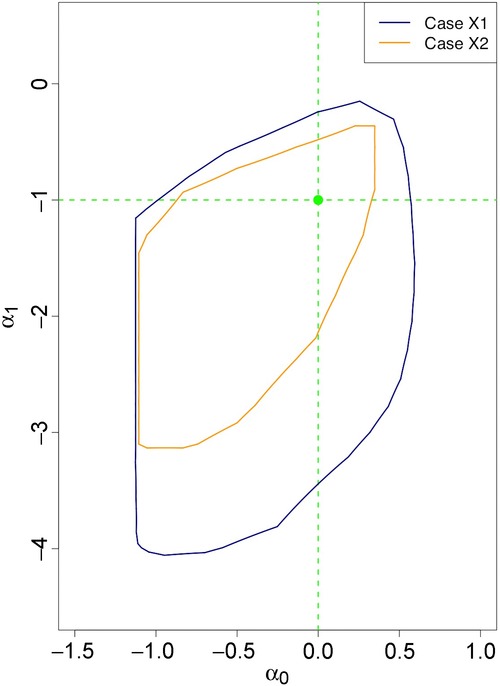
Projections of identified sets for cases X1 and X2.

Figure 1 shows the projections of the identified set in cases N1 and N2 in which *X* is endogenously determined. The probability generating value 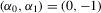 is plotted. When the parameter 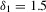 (drawn in beige, labelled Case N2), the area of the projection is smaller than when 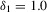 (drawn in blue, labelled Case N1). Most values in the projection when 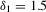 lie inside the projection obtained when 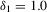, but at high values of α_0_ there is a very small region of the first projection that is not contained in the latter. Note that this can happen because even though the slope coefficient on *Z* in (4.1) is larger in the 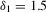 case, this does not guarantee that the quantity 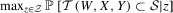 providing the lower bound of the inequalities in (4.3) is larger than in the 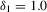 case. Figure 2 similarly illustrates projections of the identified set for cases X1 and X2 in which *X* is exogenously determined in the probability generating process. In this case, the projection of the identified set when 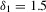 is a subset of that when 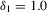. The identified sets are larger in the exogenous *X* cases, even though the predictive power of the instrument is the same as in the endogenous *X* cases. This occurs because the scale on which 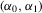 is measured differs in the two cases.^13^ Computations for both figures were implemented as described in Appendix B, with the alphahull parameter set to 5.

In all cases, the projections contain no positive values of α_1_, so the model allows one to sign α_1_ and the hypothesis 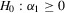 is falsifiable.

## 5. CONCLUSION

In this paper, we have provided set identification analysis for a model of binary response featuring random coefficients and potentially endogenous regressors. The regressors in question are not restricted to be distributed independently of the random coefficients. We have shown that with an IV restriction we can apply analysis along the lines of that in CRS and Chesher and Rosen et al. (2013) to characterize the identified set as those distributions that satisfy a collection of conditional moment inequalities. In our numerical illustrations of Section 4, there are 32 such inequalities, one for each core-determining set, which hold conditional on any value of the instrument. While our focus was on identification, recently developed approaches for estimation and inference based on such characterizations, such as those of Andrews and Shi et al. (2013) and Chernozhukov et al. (2013), are applicable. In some settings, the number of core-determining sets in the full characterization can be quite large, necessitating some care in choosing the number to employ in small samples. Issues that arise as a result of many moment inequalities have been investigated in an asymptotic paradigm by Menzel et al. (2009). With discrete endogenous variables having finite support, the number of conditional moment inequalities can be large, but is necessarily finite, and future research on finite sample approximations for inference and computational issues is warranted.

We have provided numerical illustrations of identified sets under particular data-generation processes. We have given an overview of the computational approach we used for computing these identified sets, and details are set out in Appendix B.

Although our computational approaches are adequate for the examples considered, we have no doubt that they can be improved, either by developing more efficient implementations, or by devising new computational approaches altogether. Nonetheless, the illustrations serve to demonstrate the feasibility of computing identified sets in one particular setting in the general class of IV models studied in Chesher and Rosen et al. (2013). These IV models can admit high-dimensional unobserved heterogeneity, for example through a random-coefficients specification such as the one studied in this paper.

## Footnotes

1 Similarly, the random intercept β_0_ can be easily removed from the analysis by restricting  throughout.

2 The model is incomplete because there is no specification for the determination of *X* given exogenous variables *Z* and unobserved heterogeneity β. Thus, for any realization of , each *x* on the support of *X* is a feasible realization of *X*. However, the triangular structure used in the control function approach implies a unique value of *X* for any realization of exogenous variables and unobservables.

3 In a binary choice model, the presence of unobserved, additively separable, alternative-specific utility shifters can be subsumed into the threshold-crossing specification, and so is unnecessary.

4 If  is bounded, then the absolute continuity condition should be understood to be required to hold with respect to the uniform measure on .

5 Such normalizations are not strictly required when allowing for set identification, but it is wise to impose them in order to enable comparison of set and point identifying models.

6 For further details and results regarding selections of random sets, see, e.g., Section 1.2 of Molchanov et al. (2005).

7 The requirement that  lives on  is innocuous. If this were not the case, then one could simply redefine the initial probability space as the product of  and the space on which  lives.

8 If Supp is uncountable, then the collection of all such test sets will include uncountable unions. If Supp is discrete and finite, then there are only finitely many possible unions of sets , and only finite unions are required.

9 The restrictions used to ensure point identification include the requirements that for some fixed , , and that the distribution of *W* has an absolutely continuous component with everywhere positive density. Our characterizations of the identified set, given by (3.6) and (3.7) in the case of only exogenous covariates, do not require these restrictions.

10 The restriction to cases where Supp is discrete is not essential but simplifies the exposition. An identical characterization of required test sets  can be shown more generally by referring back to (3.2) appearing in (3.4) and making use of the absolute continuity of  from Restriction 2.3.

11 In our R programs (R Core Team, 2012), the bivariate normal orthant probabilities are calculated using the pmvnorm program provided in the mvtnorm package (Genz et al., 2012), which implements computation of multivariate normal and *t* probabilities from Genz and Bretz et al. (2009).

12 In this section, we write  in place of  because there are no exogenous covariates *W* in this model.

13 The scale difference arises because of the differential variability of the index  in (2.1) as measured by the conditional variance given *X* and *Z*. Calculations using simulated values of the unobservables show that this is larger at every value of *X* and *Z* in the exogenous *X* case.

## APPENDIX A: PROOF OF THEOREM 3.1

**Proof of Theorem 3.1** Following the same steps as in the proof of Theorem 1 of CRS applied to the random set  and exogenous variables  in place of  and instruments *Z* in the notation of that paper, we obtain

where  denotes all closed subsets of . Then, the application of Theorem 2 of CRS, specifically part (i), further gives that  above can be replaced with unions of members of the support of . Then, using the same reasoning as in Lemma 1 of Chesher and Rosen et al. (2012), it follows that when considering probabilities conditional on ,  can be replaced by unions of elements of the conditional support of  given the realization of the exogenous variables, namely . The representation

follows from the equivalence

that for all , , and for all ,

## APPENDIX B: COMPUTATIONAL DETAILS

In this appendix, we provide computational details for the numerical illustrations of Section 4 not provided in the main text.

### B.1. Calculation of probabilities

Each set  in the collection  is the union of one or more contiguous cones centred at the point , which we refer to as elementary cones. The slopes of the rays defining the cones are determined entirely by the values of the points of support of *X*. In the case , there are eight such cones. For each value of  encountered, we calculate the probability mass supported on each of the eight cones by a bivariate normal density function with mean (0, 0) and variance matrix entirely determined by . The probability mass supported by a particular set  at the value of θ is obtained by adding the masses on the appropriate cones. Thus, we are able to compute the probability mass  allocated to each of the 32 core-determining sets by summing probabilities obtained for the eight elementary cones.

The probability masses on each elementary cone are obtained by numerical integration after re-expressing the integrand in polar coordinates. In our R code, the numerical integrations are carried out by using the adaptIntegrate function provided in the cubature package (Johnson, 2011). We have also programmed this calculation in mathematica using the NIntegrate function and an integrand, which is the appropriate bivariate normal density function with values outside the cone of interest set to zero using the Boole function. We obtained very close agreement.

The numerical integrations are necessarily computationally burdensome and some inaccuracy is inevitable, which has a knock-on effect on the determination of membership of projections.

### B.2. Calculation of projections

First approximations to the -projections of identified sets were obtained by evaluating over a coarse grid of values of . Refinements were then obtained by using a bisection procedure to search down a sequence of rays defined by angles , each passing through the probability-generating value , which is known to lie in the projection. Each ray was stepped along until a value of  outside the projection was found. A value midway between this value and the last value found in the projection was then evaluated for membership of the projection. By repeated bisection, a good approximation to the position of the boundary of the identified set along the ray under consideration was obtained. Sweeps were also made in directions parallel to the α_0_ and α_1_ axes to refine the boundary approximations in areas where it was relatively non-linear. These were helpful in confirming the near convexity of the projections, which is sufficient for our bisection-along-rays procedure to give a good view of the entire boundary.

The objective function minimized in (4.5) when determining membership of the identified set is not very well behaved. There are points at which it is not differentiable and there appear to be some places in which there are small jump discontinuities. One difficulty is that the terms  depend upon eight numerical integrals of bivariate normal density functions, and the inaccuracy in calculating these affects the computation of the minimum in (4.5). The effect is likely to be dependent on the parameter value  being considered.

There is plenty of scope for improvement in the numerical procedures employed here. In particular, a very small further investment would deliver a much more efficient method of searching down a ray for an initial point outside the identified set. The method we use relies on the near convexity of the projection

There were a few cases in which isolated points appeared to be in the projections. These were examined individually and, in most cases, by choosing different starting points for the parameters  of the minimization, the points were found on recalculation not to be in the projection. The remaining isolated points had a minimized value of the objective function in (4.5) that was very close to zero. The graphs of the identified set shown here were produced by assigning points with values of the minimized objective function less than 0.001 to the projection.

### B.3. Graphics

The projections calculated using our approximations are not convex although the departures from convexity are quite small. We do not know whether the projections are, in fact, convex with the non-convexity arising because of approximation errors. In this circumstance, it seems unwise to draw boundaries of projections as the convex hulls of the points calculated to lie in the projections, although in fact there is not so great an error produced by proceeding in this way. The projections drawn in Figures 1 and 2 are alpha-convex hulls calculated using the ahull function provided in the R package alphahull (Pateiro-Lopez and Rodriguez-Casal, 2009) with the alphahull parameter set equal to 5. We experimented with different values of this parameter and found that the differences in the illustrations were minute.

## APPENDIX C: IDENTIFICATION IN EXAMPLE 2.1 WITH EXOGENOUS *X*

Consider the setting of Example 2.1, but where, in addition, *X* is restricted to be exogenous. Here, we show that the Gaussian random-coefficients probit model is point identifying in this case.

The model stipulates that

and, with ,

where  is the variance of *U*_1_.

It follows that

and thus

(C.1)

where

is point identified under Restriction 2.1.

The Gaussian random-coefficients probit model with exogenous *X* is point identifying if there is a unique admissible solution for  to the system of equations generated by (C.1) when *x* takes all values in the support of *X*. Admissible solutions are real-valued with .

In our numerical illustrations,  and the parameter values employed are

Thus,  and

(C.2)

Setting  in (C.1) delivers

Using this and (C.2) and setting  in (C.1) delivers

(C.3)

Setting  and then  in (C.1) gives the following pair of equations in :

(C.4)

(C.5)

Solving (C.3)–(C.5), we have the unique solution , from which it follows that , and thus  is point identified.
